# Muscle Health and Prognosis in Patients With Cancer: New Insights

**DOI:** 10.1002/jcsm.70292

**Published:** 2026-04-30

**Authors:** Emanuele Cereda, Amanda Casirati, Maria Cristina Gonzalez, Nilian Carla Souza, Carla M. Prado, Katherine L. Ford, Silvia Fernandes Mauricio, Maria Isabel Toulson Davisson Correia, Paolo Pedrazzoli, Riccardo Caccialanza

**Affiliations:** ^1^ Clinical Nutrition and Dietetics Unit Fondazione IRCCS San Matteo Pavia Italy; ^2^ Postgraduate Program in Nutrition and Food Federal University of Pelotas Pelotas Rio Grande do Sul Brazil; ^3^ Brazilian National Cancer Institute Rio de Janeiro Brazil; ^4^ Department of Agricultural, Food and Nutritional Science University of Alberta Edmonton Alberta Canada; ^5^ Department of Clinical and Social Nutrition Universidade Federal de Ouro Preto Ouro Preto Brazil; ^6^ Department of Surgery Universidade Federal de Minas Gerais Belo Horizonte Brazil; ^7^ Medical Oncology Unit, Fondazione IRCCS Policlinico San Matteo and Department of Internal Medicine University of Pavia Pavia Italy; ^8^ Department of Oncology and Hematology‐Oncology University of Milan Milan Italy

**Keywords:** cancer, handgrip strength, mortality, muscle mass, muscle radiodensity, sarcopenia

## Abstract

**Background:**

Reduced muscle mass and impaired composition have each been independently associated with worse outcomes in patients with cancer. However, emerging evidence suggests that reduced muscle strength—namely, dynapenia—may be particularly important for prognostication, as it is easier to assess in clinical practice compared to muscle mass. Importantly, muscle mass and composition—as assessed with computed tomography images—may not fully capture key physiological changes and/or reflect whole‐body alterations, particularly in patients who remain within the normal range. We investigated the predictive power for mortality of low muscle mass and impaired composition, weight loss (WL) and low strength, as well as their combination, in a cohort of patients with cancer.

**Methods:**

Baseline data on muscle mass and radiodensity (Hounsfield units [HU]) at the L3 level—assessed using computed tomography—along with 6‐month unintentional WL (relevant if ≥ 10% of usual body weight) and muscle strength by handgrip were pooled for 477 patients with cancer (59.1% male, mean age 61.2 ± 12.8 years) from studies conducted in Brazil, Canada and Italy. Patients were categorized by sex and body mass index–specific cutoffs for low skeletal muscle mass index, low skeletal muscle radiodensity, WL ≥ 10% and low handgrip strength. Patients were followed for at least 12 months until death or censoring.

**Results:**

During a median follow‐up of 43 months (IQR: 28–83), 188 patients died. Kaplan–Meier analysis showed no survival differences for low skeletal muscle index or low radiodensity, regardless of handgrip strength. Only WL ≥ 10% consistently identified patients with poorer prognosis, independently of low handgrip strength. Fully adjusted Cox's regression models showed an independent association only with WL (HR = 1.56 [95% CI: 1.12;2.16]; *p* = 0.008) and low handgrip strength (HR = 2.07 [95% CI: 1.47;2.92]; *p* < 0.001), as well as an increased risk for all low handgrip strength/%WL categories. Mortality risk increased across all low handgrip strength/%WL categories. Among the eight risk groups combining low skeletal muscle mass index, WL ≥ 10% and low handgrip strength, only those including WL ≥ 10% and low handgrip strength were significantly associated with higher mortality. Low skeletal muscle mass index contributed to a worse prognosis only when combined with both WL ≥ 10% and low handgrip strength. Similar results were observed when skeletal muscle radiodensity was used in replacement of skeletal muscle mass index.

**Conclusions:**

In patients with cancer, muscle strength and WL were stronger survival predictors than muscle mass and composition, reinforcing their relevancy as easily assessed key markers of muscle health.

## 1 Introduction

Body composition—particularly in the context of the overweight/obesity epidemic—has become a key focus in nutritional assessment. Current guidelines advocate for its evaluation in all patients at nutritional risk, with particular emphasis on those with cancer due to its strong association with prognostic implications [1, 2, 3, 4, 5, 6]. In this setting, reduced skeletal muscle mass and composition—namely, radiodensity—have been independently associated with reduced survival, quality of life and increased risk of chemotherapy‐related toxicity [7, 8, 9, 10, 11, 12].

While much of previous research to date has equated sarcopenia with low muscle mass in cancer, recent evidence highlights muscle function as a superior prognostic indicator. In the context of sarcopenia guidelines, it is now internationally recognized that in chronic diseases, both muscle mass and strength should be assessed, with strength being the primary consideration [3, 13]. Notably, in the context of cancer, studies demonstrated that impaired muscle function is independently associated with worse outcomes (e.g., mortality, dose‐limiting toxicity and perioperative complications), even in patients with preserved lean/muscle mass. However, the prognostic risk is further amplified when both impairments coexist [4, 14, 15, 16, 17, 18].

Weight loss (WL) is another key prognostic marker in patients with cancer, closely related to malnutrition and/or cachexia [19]. Unintentional WL in this population is heterogeneous but consistently associated with unfavourable changes in body composition—particularly reductions in muscle mass and fibre type—primarily driven by systemic inflammation and reduced food intake [5, 19, 20, 21]. Unfortunately, muscle mass and composition—as assessed with computed tomography (CT) images—do not account for important relative changes, particularly when patients still remain within the range of normality because of a high original muscle mass reserve. Conversely, individuals with a lifelong low muscle reserve due to constitutional thinness may still maintain good functional capacity and performance status. To address these complexities, a grading system for nutritional risk incorporating body mass index (BMI) and WL has been proposed [22].

Extensive discussions have focused on the selection of muscle mass versus function as key outcomes. Functional measures have been more consistently prioritized in sarcopenia research in older adults, as outcomes tend to follow function more closely [13, 23, 24]. Furthermore, intervention studies addressing muscle health have shown a greater improvement in functional and performance endpoints than body composition, particularly in the short term [25, 26].

In this context, we conducted a secondary analysis of prospectively collected data from multiple studies to assess the independent role of established muscle‐related prognostic parameters—namely, mass, quality and strength—in predicting mortality in patients with cancer.

## 2 Subjects and Methods

### 2.1 Participants

Data were collected from patients with cancer from Brazil, Canada and Italy. They were consecutively included in previous observational (NCT02901132) [27, 28, 29] and interventional studies (NCT02055833, NCT02065726 and NCT02788955) [30, 31, 32]. Studies were conducted at the Brazilian National Cancer Institute (Rio de Janeiro, Brazil), the Hospital das Clínicas/Universidade Federal de Minas Gerais (Belo Horizonte, Brazil), the University of Alberta, (Edmonton, AB, Canada) and the Fondazione IRCCS Policlinico San Matteo (Pavia, Italy).

For all included studies, common inclusion criteria were age ≥ 18 years, a diagnosis of neoplastic disease (either solid or hematologic), complete data on relevant clinical features (cancer type, stage, performance status and anthropometry), muscle‐related exposure (handgrip strength and muscle mass evaluation by CT) and survival (at least 1‐year follow‐up).

### 2.2 Assessments

For all participants, in addition to demographic data, the following information was collected at baseline upon inclusion in the original study:

1. Clinical: cancer site and stage (American Joint Committee on Cancer stage groupings), treatment status (pre‐treatment or treatment ongoing or treatment completed) and Eastern Cooperative Oncology Group (ECOG) performance status. Specifically, this last one is a scale used to assess a patient's level of functioning and ability to tolerate therapies, particularly in oncology. Values range from 0 to 4, where 0 indicates that the patient is fully functional and asymptomatic and 4 indicates that the patient is bedridden (available at https://ecog‐acrin.org).
2. Anthropometry: body weight and height were measured (to the nearest 0.1 kg and 0.5 cm, respectively) to derive BMI, calculated as weight (kg)/height (m^2^); percentage of 6‐month unintentional weight loss (%WL) was also calculated and considered a relevant prognostic factor if ≥ 10% [1, 33].
3. Muscle mass and composition: based on correlation with whole‐body skeletal muscle [34], skeletal muscle mass area (SM, in cm^2^)—encompassing psoas, erector spinae, quadratus lumborum, transversus abdominus, external and internal obliques as well as rectus abdominus muscles—was measured using images from the third lumbar vertebra (L3) through the Slice‐O‐Matic software (Version 5.0; Tomovision, Montreal, Quebec, Canada) using attenuation thresholds of −29 to 150 Hounsfield units (HU) to identify skeletal muscle [35]. Skeletal muscle index was calculated (muscle area [cm^2^]/height [m]^2^) accordingly. Muscle composition was also determined according to radiodensity (skeletal muscle density—attenuation in HU). Therefore, participants were stratified into sex and country‐specific quantiles of the distribution of each variable. The lowest quartiles were taken as high‐risk conditions. All images were collected within 60 days prior the assessment.
4. Muscle strength: digital or hydraulic hand dynamometry (DynEx, Akern/MD Systems [Florence, Italy] in Italians; Jamar Plus Digital Hand Dynamometer [South RD Hilton, South Australia, Australia] in Brazilians; Jamar Hydraulic Hand Dynamometer [Sammons Preston Rolyan, Bolingbrook, IL, USA] in Canadians) was used to assess handgrip strength in the dominant hand. Patients were tested in triplicate in a sitting position with the shoulder adducted and neutrally rotated, the elbow flexed at 90° and the forearm and wrist in a neutral position. Low handgrip strength was defined as a value < 2 or 2.5 standard deviations (SDs) from the reference young population: for Brazilians, 29.7 and 16.2 kg for males and females, respectively [36]; for White‐Caucasians, 27 and 16 kg for males and females, respectively [37]. Three measures were obtained from each patient and the largest value was used in the analysis.

### 2.3 Outcome Ascertainment

Patients were actively followed for at least 12 months until death or censoring (date of last contact). The following follow‐up methods were used to ascertain vital status: medical records, in‐office visits, inquiries by mail or telephone to participants or proxy respondents and eventual linkage to municipal registries.

### 2.4 Statistical Analysis

Descriptive statistics were provided for both categorical (count and percentage) and continuous variables (mean and SD) or median interquartile range (IQR). Between‐country comparisons were performed using Fisher's exact test (categorical) and general linear models (continuous). The reverse Kaplan–Meier method was used to calculate median follow‐up, and survival curves were provided for group strata according to low handgrip strength in combination with either low skeletal muscle index, low muscle density or %WL. Then, multivariable models (Cox's regression) were used to evaluate: (1) the independent association of low skeletal muscle index, low muscle density, low handgrip strength, WL (≥ 10%) and mortality; (2) the association between mortality and (I) low handgrip strength/low skeletal muscle index strata, (II) low handgrip strength/low muscle density strata and (III) low handgrip strength and %WL strata. The hazard ratio (HR) and 95% confidence interval (95% CI) were computed accordingly. All models were adjusted for relevant noncollinear confounders (sex, age, ECOG performance status, disease stage and setting of treatment [coded as pre‐treatment vs. ongoing vs. posttreatment]), and intracentre correlation (inhomogeneity in variance) was accounted using Huber–White robust standard errors. Data were analysed using the software STATA 16.1 (Stata Corporation, College Station, TX, USA), setting statistical significance to a two‐sided *p* level of < 0.05.

### 2.5 Ethics

This is a secondary analysis of data from multiple studies for which the evaluation of survival had already been approved by each institutional ethics committee/board. Written informed consent was obtained from every patient.

## 3 Results

In total, 477 patients were included in the analyses (Italians, *n* = 155; Brazilians, *n* = 275; Canadians, *n* = 47). Reasons for exclusion were lost to follow‐up, *n* = 1; missing CT scan data, *n* = 297. A description of the clinical and nutritional characteristics of the study population by country of origin is provided in Tables 1 and 2. The frequencies of key muscle‐related features were low skeletal muscle index, 24.7% (*n* = 118); low muscle density, 24.9% (*n* = 119); low handgrip strength, 31.7% (*n* = 151); WL ≥ 10%, 32.3% (*n* = 154). Significant between‐country differences were found in clinical and demographic features, as well as in all muscle health parameters. Generally, females presented lower skeletal muscle index, muscle density and handgrip strength than males regardless of the country, with the exception of muscle density in Italian females, which was slightly higher than in males. Italian participants had lower handgrip strength, while lower muscle density was observed in Brazilian patients.

**TABLE 1:** Features of the study cohort by country of origin.

Characteristic	Whole cohort (*N* = 477)	Brazil (*n* = 275)	Canada (*n* = 47)	Italy (*n* = 155)	*p* ^a^
**Sex** (male), *n* (%)	282 (59.1)	149 (54.2)	28 (59.6)	105 (67.7)	0.023
**Age** (years), median (SD)	61.2 (12.8)	60.8 (11.9)	57.4 (10.2)	63.1 (14.6)*	0.022
**Body mass index** (kg/m^2^), mean (SD)	25.2 (5.4)	26.5 (5.2)	27.6 (5.5)	22.1 (4.2)*	< 0.001
**6‐Month weight loss** (%), mean (SD)	7.8 (7.9)	5.3 (7.3)	6.5 (5.5)	12.2 (7.1)*	< 0.001
**≥ 10%**, *n* (%)	154 (32.3)	50 (18.2)	9 (19.1)	95 (61.3)	< 0.001
**Setting of treatment**, *n* (%)					< 0.001
***Pretreatment***	230 (48.2)	101 (36.7)	47 (100)	82 (52.9)	
***Treatment ongoing***	137 (28.7)	64 (23.3)	—	73 (47.1)	
***Posttreatment***	110 (23.1)	110 (40.0)	—	—	
**Cancer site**, *n* (%)					< 0.001
***Gastrointestinal***	375 (74.6)	275 (100)	47 (100)	34 (21.9)	
***Head and neck***	52 (10.9)	—	—	52 (33.5)	
***Urogenital***	22 (4.6)	—	—	22 (14.2)	
***Haematologic***	10 (2.1)	—	—	10 (6.5)	
***Lung***	13 (2.7)	—	—	13 (8.4)	
***Others***	24 (5.0)	—	—	24 (15.5)	
**Cancer stage**, *n* (%)					< 0.001
***I***	27 (5.7)	20 (7.3)	0 (0.0)	7 (4.5)	
***II***	81 (17.0)	58 (21.1)	4 (8.5)	19 (12.3)	
***III***	138 (28.9)	84 (30.5)	30 (63.8)	24 (15.5)	
***IV***	231 (48.4)	113 (41.1)	13 (27.7)	105 (67.7)	
**ECOG performance status**, *n* (%)					< 0.001
***0–1***	428 (89.7)	266 (96.7)	38 (80.9)	124 (80.0)	
***2***	49 (10.3)	9 (3.3)	9 (19.1)	31 (20.0)	

Abbreviations: ECOG, Eastern Cooperative oncology Group; SD, standard deviation.

^a^ For comparison between groups according to general linear model (continuous variables; *significantly different from the other groups by post hoc comparisons) or Fishers exact test (categorical variables) as appropriate.

**TABLE 2:** Muscle health features of the study cohort by country of origin.

Characteristic	Whole cohort (*N* = 477)	Brazil (*n* = 275)	Canada (*n* = 47)	Italy (*n* = 155)	*p* ^d^ for country for sex [for interaction]
**Skeletal muscle area at L3** (cm^2^), mean (SD)	117.7 (38.6)	111.3 (40.8)	143.0 (37.0)	121.4 (31.0)	< 0.001
**Males**, mean (SD)	142.2 (36.4)	129.9 (39.4)	165.1 (30.2)	131.7 (29.0)	< 0.001
**Females**, mean (SD)	99.8 (28.1)	89.9 (30.2)	110.5 (16.2)	99.7 (22.7)	[0.11]
**Skeletal muscle index** (cm^2^/m^2^), mean (SD)	42.9 (12.3)	42.0 (13.9)	48.3 (9.6)	42.9 (9.4)	0.009
**Males**, mean (SD)	47.9 (12.0)	46.2 (13.7)	52.7 (9.0)	44.8 (9.3)	< 0.001
**Females**, mean (SD)	39.2 (11.1)	36.9 (12.5)	41.8 (6.0)	38.9 (8.2)	[0.27]
**Low SMI** ^**a**^, *n* (%)	118 (24.7)	68 (24.7)	12 (25.5)	38 (24.5)	
**Skeletal muscle density** (HU), mean (SD)	35.7 (10.2)	32.2 (8.4)	39.5 (9.9)	40.8 (10.6)	< 0.001
**Males**, mean (SD)	38.7 (9.7)	34.8 (8.1)	40.7 (8.8)	40.6 (10.9)	0.013
**Females**, mean (SD)	35.9 (10.3)	29.0 (7.8)	37.7 (11.5)	41.2 (9.9)	[0.004]
**Low SMD** ^**b**^, *n* (%)	119 (24.9)	69 (25.1)	12 (25.5)	38 (24.5)	
**Handgrip strength** (kg), mean (SD)	27.1 (10.6)	29.3 (9.9)	31.6 (11.1)	21.9 (9.6)	< 0.001
**Males**, mean (SD)	33.1 (10.2)	35.6 (8.3)	38.4 (9.3)	25.2 (9.2)	< 0.001
**Females**, mean (SD)	19.5 (6.1)	21.8 (5.2)	21.7 (3.5)	14.9 (5.9)	[0.021]
**Low strength** ^**c**^, *n* (%)	151 (31.7)	55 (20.0)	3 (6.4)	93 (60.0)	

Abbreviations: SD, standard deviation; SMD, skeletal muscle density; SMI, skeletal muscle index.

^a^ Defined as follows: for Brazilian, SMI < 30.3 cm^2^/m^2^ [F] and < 40.3 cm^2^/m^2^ [M]; for Canadians, SMI < 37.5 cm^2^/m^2^ [F] and < 47.1 cm^2^/m^2^ [M]; for Italians, SMI < 33.5 cm^2^/m^2^ [F] and < 38.4 cm^2^/m^2^ [M].

^b^ Defined as follows: for Brazilian, SMD < 23.1 [F] and < 28.4 [M]; for Canadians, SMD < 30.4 [F] and < 33.8 [M]; for Italians, SMD < 32.3 [F] and < 32.2 [M].

^c^ Defined as follows: for Brazilian, HG < 29.7 kg [M] and < 16.2 kg [F]; for Canadians and Italians, HG < 27 kg [M] and < 16 kg [F].

^d^ For comparison between countries and sex according to general linear model (continuous variables).

**FIGURE 1:**
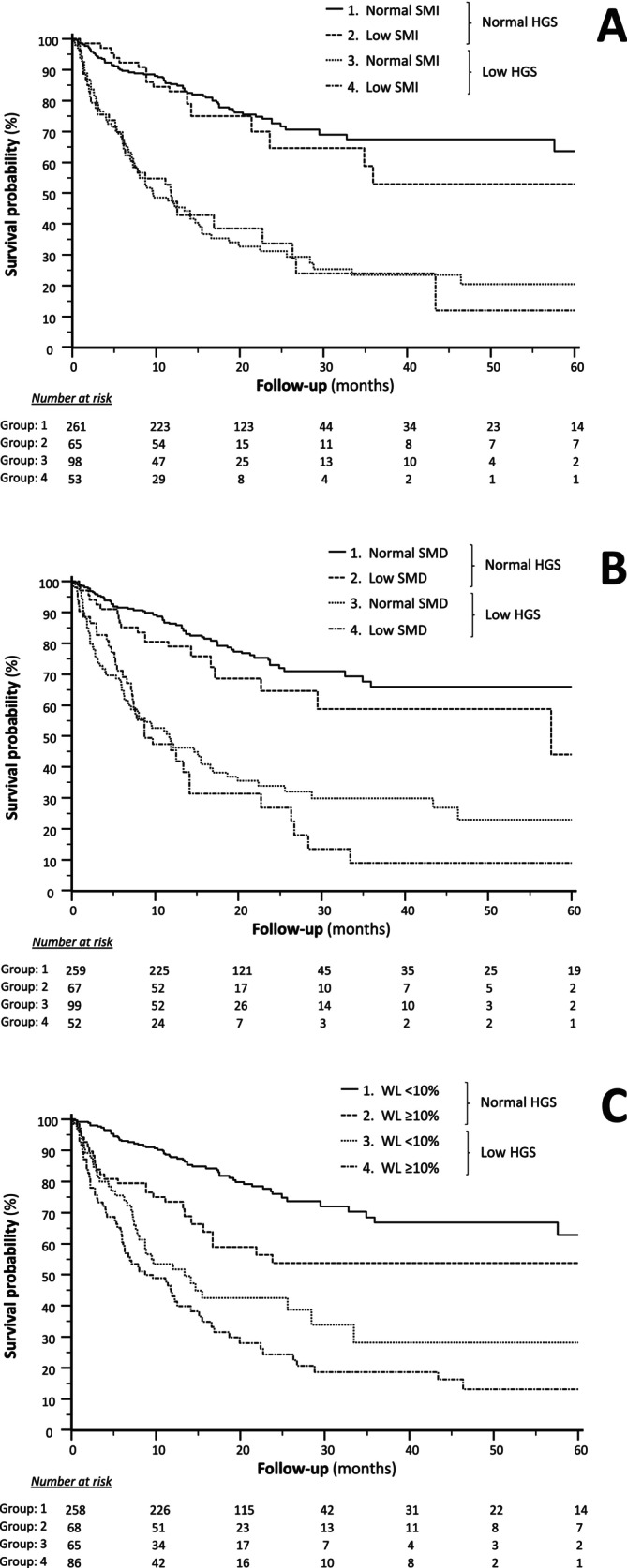
Cumulative survival curves (follow‐up truncated at 60 months) across population strata obtained by the combination of: (I) low handgrip strength (HGS) and low skeletal muscle index (SMI) (*Panel A*), (II) low handgrip strength and low skeletal muscle density (SMD) [*Panel B*] and (III) low handgrip strength and weight loss (WL) ≥ 10% (*Panel C*).

Figure 1 shows the survival curves according to the combination of muscle parameters and handgrip strength. After a median follow‐up of 43 months (IQR: 28–83), 188 patients had died (39.4%). We observed that low handgrip strength/low skeletal muscle index, low handgrip strength/low muscle density and low handgrip strength/%WL were all associated with mortality (all *p* < 0.001). However, regardless of the absence or presence of low handgrip strength, low skeletal muscle index and low muscle density were not significantly associated with survival differences. Only WL ≥ 10% independently distinguished patients with poorer prognosis, irrespective of handgrip strength status.

These findings were further supported by multivariable Cox's regression models, which identified independent associations only with WL and low handgrip strength, along with a significantly increased risk across all low handgrip strength/%WL categories (Table 3). Moreover, when low skeletal muscle index, WL ≥ 10% and low handgrip strength were combined into eight risk categories, only those including WL ≥ 10% and low handgrip strength were associated with increased mortality compared to the reference (normal handgrip strength, normal skeletal muscle index and WL < 10%). Notably, low skeletal muscle index significantly worsened prognosis only when combined with both low handgrip strength and WL ≥ 10% (Table 4). Similar results were observed when low muscle density was used for stratification in replacement of low skeletal muscle index.

**TABLE 3:** Coxs regression analysis of different risk factors and of risk categories derived by their different combination.

Risk strata	HR	95% CI	*p*	Harrells C‐index
***Model 1*** ^***a***^				0.78
Low SMI	1.26	0.89–1.79	0.19	
Low SMD	1.21	0.86–1.71	0.27	
WL ≥ 10%	1.56	1.12–2.16	0.008	
Low HGS	2.07	1.47–2.92	< 0.001	
***Model 2*** ^***a***^				0.77
Normal SMI‐normal HGS (reference)	1.00	—	—	
Low SMI‐normal HGS	1.45	0.85–2.47	0.17	
Normal SMI‐low HGS	2.53	1.75–3.66	< 0.001	
Low SMI‐low HGS	3.30	2.11–5.14	< 0.001	
***Model 3*** ^***a***^				0.77
Normal SMD‐normal HGS (reference)	1.00	—	—	
Low SMD‐normal HGS	1.41	0.84–2.35	0.19	
Normal SMD‐low HGS	2.69	1.85–3.91	< 0.001	
Low SMD‐low HGS	3.00	1.90–4.73	< 0.001	
***Model 4*** ^***a***^				0.78
WL < 10%‐normal HGS (reference)	1.00	—	—	
WL ≥ 10%‐normal HGS	2.12	1.32–3.42	0.002	
WL < 10%‐low HGS	2.70	1.76–4.12	< 0.001	
WL ≥ 10%‐low HGS	3.41	2.31–5.03	< 0.001	

*Note:* Baseline cumulative hazard function [show].

Abbreviations: 95% CI, 95% confidence interval; HR, hazard ratio; HGS, handgrip strength; SMD, skeletal muscle density; SMI, skeletal muscle index; WL, weight loss.

^a^ All models were adjusted for age, sex, setting of treatment, performance status and disease stage.

**TABLE 4:** Coxs regression analysis of different risk categories derived by the combination of muscle strength, weight loss and skeletal muscle mass (Model 1) or density (Model 2).

Risk strata	*n*	HR	95% CI	*p*	Harrells C‐index
***Model 1*** ^a^					
Normal HGS, WL < 10%, normal SMI (reference)	215	1.00	—	—	0.79
Normal HGS, WL < 10%, low SMI	44	1.64	0.86–3.13	0.13	
Normal HGS, WL ≥ 10%, normal SMI	21	2.15	1.12–4.10	0.021	
Normal HGS, WL ≥ 10%, low SMI	46	2.28	1.32–3.92	0.003	
Low HGS, WL < 10%, normal SMI	46	2.95	1.83–4.77	< 0.001	
Low HGS, WL < 10%, low SMI	19	2.72	1.26–5.89	0.010	
Low HGS, WL ≥ 10%, normal SMI	52	3.19	2.01–5.04	< 0.001	
Low HGS, WL ≥ 10%, low SMI	34	4.85	2.88–8.15	< 0.001	
***Model 2*** ^a^					
Normal HGS, WL < 10%, normal SMD (reference)	209	1.00	—	—	0.79
Normal HGS, WL < 10%, low SMD	49	1.30	0.69–2.46	0.42	
Normal HGS, WL ≥ 10%, normal SMD	50	1.98	1.14–3.45	0.015	
Normal HGS, WL ≥ 10%, low SMD	18	3.66	1.64–8.14	0.002	
Low HGS, WL < 10%, normal SMD	38	2.13	1.22–3.71	0.008	
Low HGS, WL < 10%, low SMD	25	3.14	1.72–5.75	< 0.001	
Low HGS, WL ≥ 10%, normal SMD	61	4.01	2.58–6.24	< 0.001	
Low HGS, WL ≥ 10%, low SMD	27	4.36	2.47–7.71	< 0.001	

*Note:* Baseline cumulative hazard function [show].

Abbreviations: 95% CI, 95% confidence interval; HR, hazard ratio; HGS, handgrip strength; SMD, skeletal muscle density; SMI, skeletal muscle index; WL, weight loss.

^a^ Models were adjusted for age, sex, setting of treatment, performance status and disease stage.

## 4 Discussion

In our secondary analysis of a multinational cohort of patients with cancer, muscle weakness and WL emerged as the most relevant and independent prognostic factors related to muscle health. In contrast, reduced muscle mass had an additive effect only when present alongside both.

In the literature relating to ageing, the superior prognostic value of reduced muscle strength aligns with recent findings and the sarcopenia diagnostic algorithm, which prioritizes muscle function as the primary assessment criterion [3, 13, 38]. However, reduced muscle mass in cancer is distinct from sarcopenia, although the two can coexist [5, 39]. The cancer literature has primarily focused on assessing muscle mass and composition rather than strength. However, muscle weakness has been independently associated with various outcomes and has been shown to worsen prognosis further when combined with low fat‐free or skeletal muscle mass [4, 14, 15, 16, 17, 18, 40]. Discrepancy from the literature regarding the prognostic outcome of each of these muscle health markers could be due to several factors, such as differences in study populations, measurement variability and methodological differences in statistical models, among others.

Interestingly, reduced lean/muscle mass seems to have a more limited impact in the absence of poor muscle function [4, 14, 16]. The relationship between muscle function and mass is complex. Impaired muscle function—though multifactorial and likely reflecting the inflammatory burden from the early stages of disease—can manifest before substantial muscle catabolism occurs [S1–S3]. Accordingly, the limited sensitivity of upper body strength in detecting cachexia and muscle wasting is not surprising [S4, S5], especially given the heterogeneous incidence of these two conditions across different cancer types. From this perspective, WL and low handgrip strength may be more capable of capturing relevant and detrimental changes in muscle health, thereby providing stronger prognostic value. While WL alone does not distinguish between fat and lean mass losses, we can speculate that it may still be superior to muscle mass and composition assessments. This is because CT image measures: (1) fail to identify at‐risk patients who start with a high muscle mass reserve and remain within the normal range despite significant muscle loss/deterioration and (2) do not discriminate fit patients with a naturally low muscle reserve due to constitutional thinness. Furthermore, the combination of WL and low handgrip strength aligns more closely with the definition of cachexia [19] and mirrors findings in other chronic disease models—such as chronic obstructive pulmonary disease—where wasting, inflammation and muscle dysfunction drive survival outcomes, while body mass does not [S6].

The key strength of our study lies in its clinical relevance. Both WL and handgrip strength are easily assessed in routine practice with minimal training, enabling early identification of at‐risk patients who may benefit from tailored nutritional intervention. Additionally, patients themselves can observe and report changes in body weight or strength, whereas assessing muscle mass and composition requires more specialized techniques and can only be performed at specific points in the disease course. Importantly, randomized trials addressing the efficacy of anabolic agents in patients with cancer cachexia have failed to substantially increase performance, muscle function and survival despite positive effects on body and muscle mass [S7, S8].

The importance of physical functioning on prognosis appears to be substantial. Muscle‐targeted nutritional interventions in older patients with sarcopenia and undergoing rehabilitation programs have shown greater improvements in functional and physical performance endpoints than in muscle mass itself [25, 26]. Multimodal prehabilitation (exercise + nutrition) in patients undergoing abdominal cancer surgery or exercise‐based interventions before chemotherapy resulted in better prognosis (e.g., lower postoperative complications, reduced symptom burden and hospitalization rates and increased tolerance to treatment) along with improved strength and performance [S9‐S12].

However, several limitations must be acknowledged. First, despite extensive research in this field, standardized threshold values for muscle‐related features, except for %WL, remain under debate. Interestingly, in our study, low skeletal muscle index and low muscle density were only moderately associated with outcomes, suggesting that muscle‐specific strength measures—recently proposed as a more precise indicator [13, S13]—may be more appropriate. We ran several analyses—using quartiles of muscle area and density normalized for both body weight and BMI, as well as the thresholds of skeletal muscle index and muscle density proposed by Martin et al.—but all results were consistent with the ones presented herein (data not shown) [22]. Nonetheless, we should also consider that, although L3‐muscle area strongly predicts whole‐body skeletal muscle mass [34] and handgrip strength is a valuable correlate of physical performance and functional status [S14], we cannot exclude that the relationship between muscle mass loss and mortality may be muscle‐dependent and other muscle compartments would provide better predictive value. Second, our case‐mix cohort primarily included patients with gastrointestinal cancer, a population where WL is particularly prevalent due to multiple contributing factors (e.g., inflammation and nutrition impact symptoms [e.g., anorexia, dysphagia, nausea, early satiation and constipation]). This may have influenced our results. Unfortunately, our sample size did not allow for tumour‐type stratification, highlighting the need for further studies in this area. Lastly, to develop patient‐tailored interventions, we must recognize that our study was unable to identify the most relevant determinants of WL and muscle dysfunction due to the lack of specific data. Reduced food intake, inflammation and inactivity all contribute to these derangements. Particularly, as highlighted by a large meta‐analysis on the correlations between inflammatory markers and both muscle strength and muscle mass [S15], systemic inflammation is a driver of both weight loss and sarcopenia. Therefore, a more detailed characterization of at‐risk patients—based on %WL and handgrip strength—is essential to optimize treatment strategies. Future studies should address this issue.

In conclusion, reduced muscle strength and WL emerged as stronger predictors of survival than muscle mass and composition in patients with cancer. These findings emphasize their relevance as easy‐to‐assess key characteristics of muscle health. Future studies with a stronger focus on cancer types are needed to refine operational strategies and improve patient management.

## Funding

The study was funded by the Fondazione IRCCS Policlinico San Matteo and by an unconditional grant from Abbott. CMP was partially funded by Canada‘s Research Chair Program.

## Conflicts of Interest

E.C. reports consultancies and/or speaker's honoraria from Nutricia‐Danone, Nestlé Health Science, Abbott, HARG, Akern, Wunder, Baxter and Fresenius Kabi. C.M.P. has previously received honoraria and/or paid consultancy from Abbott Nutrition, Nutricia, Nestlé Health Science, Novo Nordisk, AMRA medical and Pfizer. M.C.G. has received paid consultancy and/or speaker's honoraria from Abbott Nutrition, Nutricia and Nestlé Health Science Brazil. M.I.T.D.C. reports consultancies and/or speaker's honoraria from Abbott, Baxter, Fresenius Kabi, Nutricia‐Danone and Nestlé Health Science. R.C. reports consulting and lecture fees from Astellas, Akern, Baxter, B‐Braun, Bristol‐Myers Squibb, Boehringer Ingelheim, Eli Lilly, Fresenius Kabi, Lionhealth, Nestle’ Health Science, Novartis, Nutrisens, MSD, Nutricia, Pfizer, Roche, Servier, Takeda and Viatris. The other authors declare no conflicts of interest.

Abstract (preliminary draft) presented at the European Society for Clinical Nutrition and Metabolism (ESPEN) Congress 2024 Milan, Italy (7–10 September 2024).

## Supporting information


**Data S1:** Supplementary Material.

## Data Availability

Data described in the manuscript, code book and analytic code will not be made available because a specific note was not included in the informed consent at the time of protocol approval and recruitment. Therefore, data sharing was not approved by the Ethics Committees.
