# Supplementary material for: Muscle Health and Prognosis in Patients With Cancer: New Insights
Source: J Cachexia Sarcopenia Muscle. 2026 Apr 30;17(3):e70292. doi: 10.1002/jcsm.70292 (PMC13129677; doi:10.1002/jcsm.70292)
Supplement: Supplementary file 1 — Data S1: Supplementary Material. [file JCSM-17-e70292-s001.docx]

**Supplementary Materials**

**Muscle health and prognosis in patients with cancer: new insights.**

**References**

S1. Norman K, Stobaus N, Kulka K, Schulzke J. Effect of inflammation on handgrip strength in the non-critically ill is independent from age, gender and body composition. Eur J Clin Nutr. 2014;68:155-8. doi:10.1038/ejcn.2013.261

S2. Op den Kamp CM, Langen RC, Minnaard R, Kelders MC, Snepvangers FJ, Hesselink MK, et al. Pre-cachexia in patients with stages I-III non-small cell lung cancer: systemic inflammation and functional impairment without activation of skeletal muscle ubiquitin proteasome system. Lung Cancer. 2012;76:112-7. doi:10.1016/j.lungcan.2011.09.012

S3. Jeejeebhoy KN. Nutritional assessment. Nutrition. 2000;16:585-90. doi:10.1016/s0899-9007(00)00243-4

S4. Anderson LJ, Lee J, Mallen MC, Migula D, Liu H, Wu PC, et al. Evaluation of physical function and its association with body composition, quality of life and biomarkers in cancer cachexia patients. Clin Nutr. 2021;40:978-86. doi:10.1016/j.clnu.2020.07.001

S5. Moreau J, Ordan MA, Barbe C, Mazza C, Perrier M, Botsen D, et al. Correlation between muscle mass and handgrip strength in digestive cancer patients undergoing chemotherapy. Cancer Med. 2019;8:3677-84. doi:10.1002/cam4.2238

S6. McDonald MN, Wouters EFM, Rutten E, Casaburi R, Rennard SI, Lomas DA, et al. It's more than low BMI: prevalence of cachexia and associated mortality in COPD. Respir Res. 2019;20:100. doi:10.1186/s12931-019-1073-3

S7. Ramage MI, Skipworth RJE. The relationship between muscle mass and function in cancer cachexia: smoke and mirrors? Curr Opin Support Palliat Care. 2018;12:439-44. doi:10.1097/SPC.0000000000000381

S8. Groarke JD, Crawford J, Collins SM, Lubaczewski S, Roeland EJ, Naito T, et al. Ponsegromab for the Treatment of Cancer Cachexia. N Engl J Med. 2024;391:2291-303. doi:10.1056/NEJMoa2409515

S9. Mijwel S, Bolam KA, Gerrevall J, Foukakis T, Wengstrom Y, Rundqvist H. Effects of Exercise on Chemotherapy Completion and Hospitalization Rates: The OptiTrain Breast Cancer Trial. Oncologist. 2020;25:23-32. doi:10.1634/theoncologist.2019-0262

S10. Padilha CS, Marinello PC, Galvao DA, Newton RU, Borges FH, Frajacomo F, et al. Evaluation of resistance training to improve muscular strength and body composition in cancer patients undergoing neoadjuvant and adjuvant therapy: a meta-analysis. J Cancer Surviv. 2017;11:339-49. doi:10.1007/s11764-016-0592-x

S11. Squires RW, Shultz AM, Herrmann J. Exercise Training and Cardiovascular Health in Cancer Patients. Curr Oncol Rep. 2018;20:27. doi:10.1007/s11912-018-0681-2

S12. Waterland JL, McCourt O, Edbrooke L, Granger CL, Ismail H, Riedel B, et al. Efficacy of Prehabilitation Including Exercise on Postoperative Outcomes Following Abdominal Cancer Surgery: A Systematic Review and Meta-Analysis. Front Surg. 2021;8:628848. doi:10.3389/fsurg.2021.628848

S13. Costa Pereira JPD, Prado CM, Gonzalez MC, da Silva Diniz A, Miranda AL, de Medeiros GOC, et al. Strength-to-muscle radiodensity: A potential new index for muscle quality. Clin Nutr. 2024;43:1667-74. doi:10.1016/j.clnu.2024.05.032

S14. Lunt E, Ong T, Gordon AL, Greenhaff PL, Gladman JRF. The clinical usefulness of muscle mass and strength measures in older people: a systematic review. Age Ageing. 2021 Jan 8;50(1):88-95

S15. Tuttle CSL, Thang LAN, Maier AB. Markers of inflammation and their association with muscle strength and mass: A systematic review and meta-analysis. Ageing Res Rev. 2020 Dec;64:101185.

S16. von Haehling S, Morley JE, Coats AJS, Anker SD. Ethical guidelines for publishing in the Journal of Cachexia, Sarcopenia and Muscle: update 2019. J Cachexia Sarcopenia Muscle. 2019;10:1143-5. doi:10.1002/jcsm.12501
